# Snake Venom Peptides: Tools of Biodiscovery

**DOI:** 10.3390/toxins10110474

**Published:** 2018-11-14

**Authors:** Aisha Munawar, Syed Abid Ali, Ahmed Akrem, Christian Betzel

**Affiliations:** 1 Department of Chemistry, University of Engineering and Technology, Lahore 54890, Pakistan; 2 H.E. J. Research Institute of Chemistry, (ICCBS), University of Karachi, Karachi 75270, Pakistan; abid.ali@iccs.edu; 3 Botany Division, Institute of Pure and Applied Biology, Bahauddin Zakariya University, Multan 60800, Pakistan; ahmedakrem@hotmail.com; 4 Department of Chemistry, Institute of Biochemistry and Molecular Biology, University of Hamburg, 22607 Hamburg, Germany; 5 Laboratory for Structural Biology of Infection and Inflammation, DESY, Build. 22a, Notkestr. 85, 22603 Hamburg, Germany

**Keywords:** polypeptide structure and function, therapeutic peptides, X-ray crystallography, three-finger toxins, bradykinin potentiating peptides, crotamine, kunitz-type inhibitor

## Abstract

Nature endowed snakes with a lethal secretion known as venom, which has been fine-tuned over millions of years of evolution. Snakes utilize venom to subdue their prey and to survive in their natural habitat. Venom is known to be a very poisonous mixture, consisting of a variety of molecules, such as carbohydrates, nucleosides, amino acids, lipids, proteins and peptides. Proteins and peptides are the major constituents of the dry weight of snake venoms and are of main interest for scientific investigations as well as for various pharmacological applications. Snake venoms contain enzymatic and non-enzymatic proteins and peptides, which are grouped into different families based on their structure and function. Members of a single family display significant similarities in their primary, secondary and tertiary structures, but in many cases have distinct pharmacological functions and different bioactivities. The functional specificity of peptides belonging to the same family can be attributed to subtle variations in their amino acid sequences. Currently, complementary tools and techniques are utilized to isolate and characterize the peptides, and study their potential applications as molecular probes, and possible templates for drug discovery and design investigations.

## 1. Introduction

Nature has always been an intriguing source for drug design investigations. From among the animal kingdom, insects and reptilians are interesting and valuable sources for identifying molecules with potential pharmaceutical applications [1]. To date, various bioactive proteins and peptides have been reported from the venom of different species of snakes, conus, scorpions, centipedes, lizards, spiders, sea anemones, bees and octopus [1,2,3,4,5,6,7,8,9]. In this context, venomous snakes are a focus of investigations, and a large number of pharmacologically useful molecules have already been isolated and characterized from snake venoms [10,11]. Among the approximately 3000 known snake species, only a relatively small number are venomous, mainly belonging to Viperidae and Elapidae families. Some members of Colubridae and Atractaspididae are also reported to be venomous [12].

**Figure 1:**
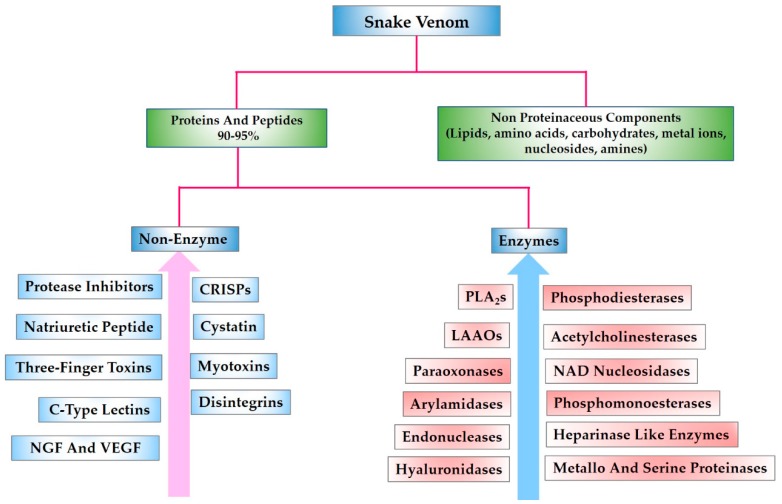
Snake venom composition.

Snake venom is a glandular secretion which snakes use to immobilize and digest their prey. It is also used as a defensive and a survival tool [13]. This lethal mixture is composed of amino acids, nucleic acids, carbohydrates, lipids, proteins and peptides [14]. Figure 1 (adapted from our own reference) illustrates the composition of snake venom [15,16].

In the present review, we focus our discussion on snake venom peptides, their classification and bioactivities. We define snake venom peptides as the group of non-enzymatic polypeptides in the venom which fold into monomeric domains and are smaller than 80–100 residues. However, variations can be found. For example, three-finger toxins and disintegrins [17,18,19] can exist as dimers. Among three-finger toxins, haditoxin [Protein Data Bank (PDB) code: 3HH7] and ҝ-bungarotoxins (PDB code: 2ABX) are non-covalent dimers, and irditoxin (PDB code: 2H7Z) and α-cobratoxin (PDB code: 2CTX) are covalent dimers [20,21]. Nonetheless, we shall not classify these dimers separately, because they bear structural and functional similarities to their monomeric counter parts.

Peptides are an interesting component of the snake venom. Although otherwise poisonous, when used in the right proportions or structurally engineered, several venom peptides can be used directly as therapeutic drugs or as drug leads, Table 1 [22,23,24,25,26]. These peptides are of great value, due to their diversified and distinct pharmacological activity, and high affinity and selectivity towards their receptors. For example, snake venom toxins have proved to be invaluable tools in determining the structure and functions of receptors. An excellent example in this regard are α-neurotoxins [27]. Snake venom peptides are mostly stable molecules, able to survive the harsh proteolytic environment of the venom gland itself. Also, it is the inherent stability of these peptides that helps them reach their target receptors, inside their prey (upon envenomation). The stability of these molecules is attained when they are recruited into the venom gland through disulfide bonds formation and/or post-translational modifications [28].

Based on the available data, snake venom peptides can be grouped into several families, discussed in Section 2. Table 1 and Table 2 present a brief structural and functional summary of these peptides.

Applying advanced analytical tools and methodologies is important to investigate and unravel the complexities of these venomous mixtures. Today, several integral approaches and complementary techniques are applied to isolate, characterize and explore the structure-function relationships of snake venom peptides. Advancements in proteomics, multidimensional chromatographic and mass spectrometric methods, requiring only small amounts of sample, have paved the way towards the discovery and characterization of these peptides. High-resolution X-ray crystallographic techniques provide insights into the three-dimensional structures of snake venom peptides and their complexes with proteins. Also, chemical and bioassays are of utmost importance in leading towards the exploration of novel functions of these biomolecules. In addition to the above-mentioned experimental methods, bioinformatics and molecular modeling approaches allow scientists to study the interaction of snake venom peptides with proteins and other complex environments and are therefore an important tool for rational drug design investigations.

## 2. Snake Venom Peptides and Their Potential Pharmacological Applications

Technological advancement and continuous interest in venomous molecules have supported the discovery of a large number of snake venom peptides. The snake venom peptides are classified into distinct families based on the structural and functional similarities in the organization of these molecules.

### 2.1. Three-Finger Toxins (3FTxs)

The 3FTxs family of polypeptides is comprised of 60–74 amino acid residues. These peptides show diverse functionalities, however, having a conserved structure. A distinct structural feature of 3FTxs is the unique fold, consisting of three loops (β-stranded), emerging from a hydrophobic globular core, Table 2. Four to five disulfide bonds are present in these 3FTxs, thereby stabilizing the three-dimensional structure. Subtle variations in the length of their loops, conformations and amino acid residues are responsible for their distinct biological functions [48]. Based on their function, these snake venom peptides can be classified into the groups described in Section 2.1.1, Section 2.1.2, Section 2.1.3, Section 2.1.4, Section 2.1.5 and Section 2.1.6.

Proteomic and transcriptomic analysis has shown that the ratio of 3FTxs can be higher relative to other toxins in the Elapidae venoms, as shown, for example, in the venom of *Naja ashei*, *Micrurus pyrrhocryptus* and *Micrurus t. tschudii* [49,50,51,52,53]. 3FTxs constitute more than 60% of the venom composition of cobra snakes [54,55,56,57], while there is a wide distribution of relative abundance of 3FTxs among different krait species, ranging from as low as 1.3% in *B. fasciatus* [58,59] to as high as 60% in *Laticauda colubrina* (a sea krait) [60,61]. The proteomic study of mamba venoms revealed that 3FTxs are also one of the most abundant toxins in their venom [50,62,63,64,65]. Proteomic studies have also shown that a combination of different 3FTxs can be present in the venom of the same species [58]. Although these peptides primarily exist in the venom of the Elapinae subfamily, however, they have also been reported for venoms of hydrophiids, colubrids, viperids and crotalids, either at the transcriptome or protein level [66,67,68].

Evolutionary studies on 3FTxs showed that these peptides evolved from genes encoding non-toxic ancestral proteins, as the three-finger scaffold can be found in other non-venom proteins [69,70]. It was reported by Fry and colleagues that three-finger neurotoxins are evolving under positive selection in line with the receptors of prey species, while the evolution of three-finger cytotoxins is limited by negative selection, as they interact nonspecifically with the cell membranes [71].

#### 2.1.1. Neurotoxins

The primary target of these toxins is the cholinergic system, and they display selectivity towards different receptor subtypes. Based on their selective binding with cholinergic receptors, they can be further classified as:

##### Curaremimetic Toxins (α-Neurotoxins or Postsynaptic Neurotoxins)

These peptides show very high selectivity, specificity and affinity towards postsynaptic nicotinic acetylcholine receptors (nAChRs). Therefore, they impede the acetylcholine neurotransmission at the skeletal muscle neuromuscular junction. The α-neurotoxins are of two types: short-chain neurotoxins (60–62 amino acids, four disulfide bridges) and long-chain neurotoxins (66–74 amino acids, five disulfide bridges). The long-chain peptide binds with higher affinity with neuronal α_7_ nAChR. This specificity of the long-chain peptide towards its target is attributed to the presence of the fifth disulfide bond.

The crystal structure of α-Bungarotoxin in complex with nAChR α_1_ revealed an unprecedented view of the interactions between the receptor and the toxin. It was determined that Arg^36^ and Phe^32^ (present in the finger II of the toxin) are the two key residues which block the aromatic cage of the receptor. The toxin also disrupts the interaction between the glycan chain on the Cys-loop and loop C, which breaks the communication between the ligand binding site and the membrane pore [72].

The α-elapitoxin, isolated from *Dendroaspis p. polylepis*, was found to have an amidated C-terminal arginine. This post-translational modification is rarely observed for snake venom three-finger toxins. Assay binding studies showed that α-elapitoxin strongly inhibits α_7_ nAChR; however, it was suggested that the C-terminal modification does not significantly affect toxin selectivity [73]. The three-finger toxin peptides have served as an excellent molecular probe in deciphering the structural and functional details of nAChR since the discovery of the iconic peptide α-bungarotoxin from the venom of a krait, *Bungarus multicinctus* [74,75]. This discovery inspired further studies in the field, and as a result, it was possible to understand various medical problems like myasthenia gravis [76]. Bungarotoxin is still a molecule of choice in delineating underlying mechanisms of many neuronal disorders. Mutation of the invariant Cys-loop Asp residue, in the acetylcholine receptor δ-subunit was identified in a patient suffering from congenital myasthenia syndrome [77]. A recent study employing the use of radioactive derivative of α-bungarotoxin suggested the possible function of neuronal nAChR in the development of Parkinson’s disease [78]. Apart from serving as molecular probes in various medical disorders, three-finger neurotoxins are also gaining attention as potential analgesics, anti-inflammatory molecules, and immunosuppressant [79]. A neurotoxin characterized from *Naja n. atra* venom in rats suppressed skin allograft rejection by inhibiting the T-cell-mediated immune response [79]. A new group of three-finger toxins (namely, Ω-neurotoxins) were also recently isolated from the venom of the *Ophiophagus hannah* snake. Interestingly, the characteristic functional residues of α-neurotoxin Oh9-1 are not conserved and interact with nAChR via distinct residues. Furthermore, unlike other α-neurotoxins, the tip of loop II is not involved; rather, the β-strand of loop II interacts with the receptor [80].

##### Muscarinic Toxins

These toxins bind to muscarinic acetylcholine receptors (mAChRs) and act as either antagonists or agonists towards various subsets of muscarinic AChR (M_1_–M_5_). An interesting example in this regard is that of MT1 (a muscarinic toxin from *Naja kaouthia*), which acts as an agonist at M_1_ and an antagonist at M_4_. This selective activity of muscarinic toxins has made them an invaluable tool for distinct biomedical investigations [81]. Other muscarinic toxins (namely MT3 and MT7) isolated from the venom of *Dendroaspis angusticeps* illustrates high specificity for M4 and M1 mAChRs, respectively [82].

##### k-Neurotoxins

The structures of these peptides are similar to long chain α-neurotoxins. They exist as dimers in contrast to other members of the 3FTxs family. They recognize only α_3_β_2_ and α_4_β_2_ nAChR subtypes and not the α_1_ type.

An interesting peptide, namely haditoxin, was isolated from *O. hannah*. Its crystal structure revealed it to be a homodimer-like kappa-neurotoxin. However, the monomeric subunits of haditoxin are structurally homologous to curaremimetic short-chain α-neurotoxins. This peptide is reported to interact with neuronal alpha_7_ nAChRs, as well as with muscle nAChRs [83].

Chemical- and site-directed mutagenesis studies have shown that different types of neurotoxins utilize a common set of amino acid residues, in addition to some other residues, such as that found at the tip of loop I in erabutoxin (*Laticauda semifasciata*) and the carboxy terminal in α-cobratoxin (*Naja kaouthia*), to distinctly interact with their target molecules [84].

#### 2.1.2. Cardiotoxins (CTXs)

**Figure 2:**
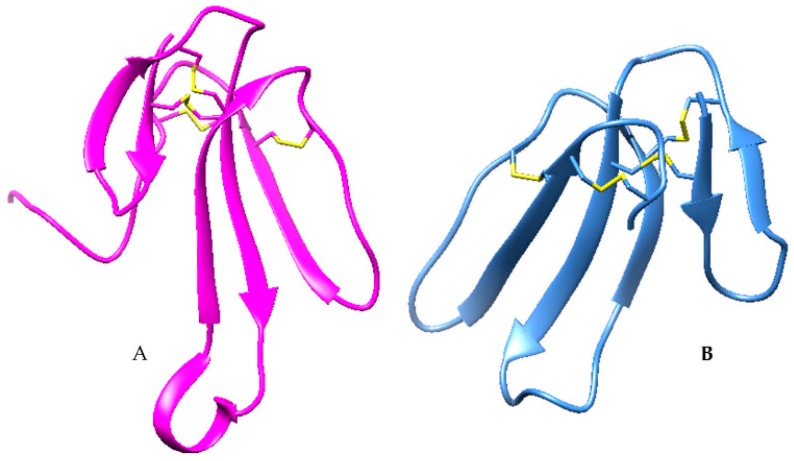
(**A**) Snake venom neurotoxin showing the extended central finger (PDB Code; 1NTN). (**B**) Snake venom cardiotoxin (PDB Code; 1ug4). Both three-finger toxins show a core of disulfide bonds from which the three-finger-like loops extend.

Cardiotoxins are the second-largest group of three-finger toxins [49]. CTXs are also known as cytotoxins because of their ability to cause lysis in many different cells [85]. They are hydrophobic, basic and, on the whole, relatively short polypeptides (60–62 amino acids). Their structure is similar to short-chain neurotoxins. In comparison to α-neurotoxins, the functional site of cardiotoxins is present at the tip of the three loops. These are generally hydrophobic amino acids, which impart amphiphilic properties to the molecule. A row of positively charged amino acids borders the hydrophobic extremities of loops I–III. This pattern of hydrophobic and cationic regions is commonly found among these cytolytic peptides. The shapes of these CTXs loops are also different to other common neurotoxins. A superposition of CTX and α-neurotoxin shows that the middle loop in the α-neurotoxin is more extended than the other two loops, as shown in Figure 2. The upper parts of both types of toxins have disulfide bridges (termed cysteine-knots) and are homologous and well superimposable [86].

Cardiotoxins are known for their membrane perturbation ability, and the extent to which these peptides can perturb the membrane is known to depend on the presence of Ser^30^ or Pro^28^ residues in loop II. Cardiotoxins are therefore classified as S-type and P-type CTXs, respectively. The P-type CTXs bind more strongly to the membranes [87]. CTXs insert into membranes containing anionic lipids and demonstrate certain characteristic features, summarized by Dubovskii et al., which differentiates them from other membrane active molecules [86]. Although until now there has been no clear evidence of whether and how CTXs interact with proteins, some indications have been suggested [88,89]. Hemachatoxin is a P-type CT isolated and crystallized from *Hemacatus hemacatus* venom. It has been suggested that loop II of this toxin is highly flexible [90].

Cytotoxins are thermostable and also show high stability towards various denaturing agents [88]. Another class of cardiotoxins known as β-cardiotoxins is known to bind to β-adrenergic receptors. Studies showed that these peptides decrease the heart rate, unlike CTXs, which increase the heart rate [91].

Snake venom cardiotoxins are presently also the focus of several cancer-inhibiting studies, and promising results have already been obtained in this regard [86,92,93]. Some studies report cytotoxins to be necrotic, while others as apoptotic agents. Cytotoxin-1 and cytotoxin-II isolated from *Naja oxiana* venom showed anticancer activity better than cisplatin (anti-cancer drug). It was also demonstrated that these toxins had a minimal effect on normal cells (i.e., MDCK), and induced their apoptotic effect in various cancer cell lines (i.e., HepG2, MCF-7, HL-60 and DU-145) via lysosomal pathway and cytosolic release of cathepsins [32]. Cardiotoxins are also known to possess antimicrobial activity. The tip and β-strand of the first finger of cardiotoxin-1 was utilized as a template to design 20 amino acid residue peptides. Interestingly, these peptides demonstrated a broad range of antimicrobial activity with low cytotoxicity and hemolytic activity towards eukaryotic cells [33].

#### 2.1.3. Acetylcholinesterase Inhibitors

These toxins are structurally similar to short-chain neurotoxins and inhibit the enzyme acetylcholinesterase at the neuromuscular junction, thereby inducing fasciculation of the muscle, and hence are named fasciculins. They block the peripheral site of the enzyme; thus, the enzyme is not able to degrade its substrate (acetylcholine) any more. Earlier studies have shown that amino acid residues present on the first and second loop of the toxin are involved in the inhibition of enzyme activity [69].

#### 2.1.4. Non-Conventional 3FTxs

These polypeptides have an additional fifth disulfide bond either in loop I or loop II. Kini and Doley described these peptides as non-conventional 3FTxs, while earlier these peptides were classified as “weak” neurotoxins [69]. Site-directed mutagenesis and NMR (nuclear magnetic resonance) studies of WTX from *Naja Kauthia* revealed that loop II of the toxin plays a key role in binding to mAChRs [82]. Candoxin, isolated from *B. candidus*, shows nanomolar-binding affinity towards both muscle and neuronal AChR. Binding to muscle AChR is easily reversible [94]. Denmotoxin and Irditoxin from colubrid venom show a relatively high binding affinity towards avian neuromuscular junction compared to that of mammals [66,69]. Mambaglins obtained from the venom of eastern green mamba demonstrated analgesic properties. In addition, these peptides also have the ability to inhibit acid-sensing ion channels [95].

#### 2.1.5. Ion Channel Blockers/Modulators

Calciseptine and FS2 isolated from the venom of black mamba are potent L-type calcium channel blockers. They bind to the 1,4-dihydropyridine binding site of the L-type calcium channel and block the passage of calcium through the channel. Structurally, they are similar to short-chain neurotoxins. It has been predicted by some studies that the functional site of these peptides is located on the III loop, between residue 42 and 47 [96,97,98].

Mambalgins (I–III), belong structurally to the non-conventional 3FTxs and specifically inhibit acid-sensing ion channels (ASICs), producing analgesic effects as strong as morphine, but with fewer side effects. Their functional domain is present in loop II, and it has been proposed that mambalgins inhibit ASICs by a pH sensor-trapping mechanism [99].

Recently, a short-chain neurotoxin designated Calliotoxin was isolated from the venom of a coral snake, *Calliophis bivirgatus*. It bears 53% sequence homology to Rho-elapitoxin-Da1b (UniProt ID: P86419). It was shown to modulate the voltage-gated sodium channel. Further studies of this novel class of 3FTxs could provide insight into the physiology and pharmacology of voltage-gated sodium channels [100]. Another recent study reported a new toxin, namely Tx7335, from *Dendroaspis augusticeps*, which activates the bacterial potassium channel KcsA. This new identified toxin has high sequence similarity to long chain neurotoxins, but greatly differs with regard to the number and positions of cysteine [101].

#### 2.1.6. Platelet Aggregation Inhibitor

Dendroaspin is known as a potent inhibitor of platelet aggregation. It contains a tripeptide sequence RGD, responsible for the adhesive function of some proteins. It renders its inhibitory activity by preventing the interaction of fibrinogen and its receptor glycoprotein. The functional site of dendroaspin is located at the tip of loop III [102,103].

### 2.2. Kunitz-Type Serine Protease Inhibitor

Kunitz-type inhibitors are a family of serine protease inhibitors found in the venoms of Elapidae and Viperidae snakes [62]. It has been suggested that they play a role in disturbing the prey’s homeostasis, mainly by interfering with the blood coagulation cascade [26]. Proteome analysis of different snake venoms has shown the relative abundance of snake venom Kunitz-type inhibitors. The Kunitz-type inhibitors represent approximately 28% of the venom proteome of the snake *Daboia russelii russelii*, found also in Pakistan [104], and approximately 16% of *Dendroaspis angusticeps* [50]. Furthermore, it is known that more than one type of Kunitz-type inhibitor can be found in the venom of a single snake species [105,106].

Evolutionary studies explain the diversification of snake venom Kunitz-type inhibitors by a positive Darwinian selection, and it was suggested that the driving force for the variability of the inhibiting loop was caused by selective pressure, driven by the presence of diverse prey proteases. With respect to functional activities, snake venom Kunitz-type inhibitors are divided into two major groups: non-neurotoxin (trypsin and chymotrypsin inhibitors) and neurotoxin (potassium and calcium blockers) snake venom Kunitz-type inhibitors [107].

Snake venom Kunitz-type inhibitors bear a structural similarity to aprotinin and consist of approximately 60 amino acids. They have three conserved disulfide bridges responsible for the stability of the molecule and two antiparallel β-strands, which are linked by a β-hairpin in the central part of the molecule. There are two helical regions, one 3_10_-helix near the N-terminus and an α-helix near the C-terminus (Table 2). Kunitz-type inhibitors interact with some serine proteases through an exposed loop in a canonical confirmation. The P1 amino acid residue is considered to be the primary reactive site, determining the specificity and extent of reactivity of the Kunitz-type inhibitor towards the serine protease. X-ray structures of Kunitz-type inhibitors in complex with serine protease revealed both the canonical binding (P3-P3′) and secondary binding loops, which represent important regions of the peptide interacting with the protease [39]. Despite the overall conserved structural scaffold, the amino acid residues in the canonical and secondary binding loop possess subtle sequence variations in these regions and thus responsible for functional diversity in snake venom Kunitz-type inhibitors [108].

The neurotoxic snake venom Kunitz-type inhibitors do not usually have serine protease inhibitory activity. However, they act as potassium and calcium channel inhibitors. A well-known example of a snake venom potassium channel blocker with Kunitz domain is dendrotoxin, isolated from mamba snakes [109]. A recent study demonstrated that dendrotoxin binds and stabilizes *eel*AChE and even enhances its activity [110], a property of dendrotoxins not described before.

The crystal structure of α-dendrotoxin (α-DTX) and its comparison with the structure of Bovine pancreatic trypsin inhibitor (BPTI) revealed subtle structural differences, which explain the inability of these toxins to form a complex with serine proteases. Differences in the amino acid sequence of the anti-protease loop of this toxin and BPTI, also indicate that required side chain interactions are different. The P1 residue of BPTI is Lys^15^, which in α-DTX is Tyr^17^, which cannot provide the same electrostatic interactions as lysine. Another residue adjacent to Tyr^17^ in α-DTX is aspartate (Asp^18^), which seems to play an important role in reducing its inhibitory properties. In BPTI and its homologues either alanine or glycine are present at this position. The Asp^18^ side chain is negatively charged, and it would be placed in an energetically uncomfortable environment, as explained by a model complex of α-DTX with trypsin. Secondly, another important residue in α-DTX is Pro^21^ (the corresponding residue in BPTI is Ile^19^), which plays an important role in preventing the binding of α-DTX with trypsin. In contrast, BPTI Ile^19^ forms a hydrophobic interaction with Tyr^39^. However, Pro^21^ of α-DTX would force a different conformation of Tyr^39^ in trypsin in order to avoid overlap considering Van der Waals distances, thus prohibiting complex formation [111].

Studies have shown that these toxins bind to the potassium channel via 3_10_-helix and β-turn, and a subtle variation in these residues allows the toxins to develop specificity for various subtypes of potassium channels [112,113,114]. Furthermore, a study reported a bifunctional peptide (i.e., BF9) possessing both Kunitz-type protease and potassium channel inhibition properties. It was suggested that the peptide uses basic residues located at its N- and C-terminus to interact with potassium channel subtype Kv1.3, and Asn^17^ (P1 site) to interact with chymotrypsin and elastase [115]. These potassium channel inhibitors are being employed to understand various physiological disorders, as painful diabetic neuropathy and thereby propose a possible treatment [116]. These Kunitz-type potassium channel inhibitors can also help to understand the structure and function of potassium channels in more detail and provide useful information for drug design investigations, targeting problems associated with the malfunctions of these channels.

Snake venom Kunitz-type serine protease inhibitors also offer promising pharmaceutical applications [117]. For example Textilinin-1 has been shown to be more effective and more specific anti-bleeding agent as compared to Trasylol [aprotinin (Bovine pancreatic trypsin inhibitor)], and demonstrate fewer side effects [118].

Textilinin-1 was isolated from the venom of *Pseudonaja textilis* [119,120]. It is a slow and tight binding inhibitor of plasmin. The peptide (TP1) isolated from *Micrurus tener tener* venom is also an inhibitor of plasmin [39,121,122]. TP1 has a molecular mass similar to Kunitz-type peptides, but its sequence is not reported till now. It was suggested that TP1 has potential therapeutic applications as antifibrinolytic agent. The inhibitory activity of TSPI (a recombinant form of a Kunitz-type inhibitor isolated from *Oxyuranus scutellats*) was investigated comparatively against 12 selected enzymes involved in hemostasis. It showed the highest inhibitory activity towards plasma kallikrein. Rusvikunin (II) and its protein complex, isolated from *Daboia r. russelii* venom inhibited the activity of trypsin, plasmin and FXa. The peptide and its complex also inhibited the fibrinogen-clotting activity of thrombin. Another Kunitz-type inhibitor (namely PIVL) isolated from *Macrovipera lebetina* venom, displayed potent anti-tumor activity through interference with integrin receptor function [26,122,123,124,125].

### 2.3. Disintegrins

Disintegrins are cysteine-rich peptides that result from the post-translational cleavage of snake venom metalloproteases (SVMPs), which are phylogenetically related with ADAMS (a disintegrin and metalloprotease). The possible function and activity of disintegrins in snake venoms is assigned to support the distribution of other toxins throughout prey tissues by binding integrins and inhibiting platelet aggregation direct upon envenomation [126]. Disintegrins are found in the venoms of mainly Crotalidae and Viperidae snakes, and constitute approximately 17% and 18% of total venom proteins, respectively [62].

Calvete and colleagues addressed the evolutionary pathways of disintegrins in detail, and concluded that different disintegrin subfamilies evolved from a common ADAM scaffold, and that structural diversification of disintegrins occurred through disulfide bond engineering and minimization of genomic and protein structures [17,127]. Furthermore, Kini has recently described that exonization and intronization plays an important role in the evolution of disintegrin/metalloprotease genes [128].

**Figure 3:**
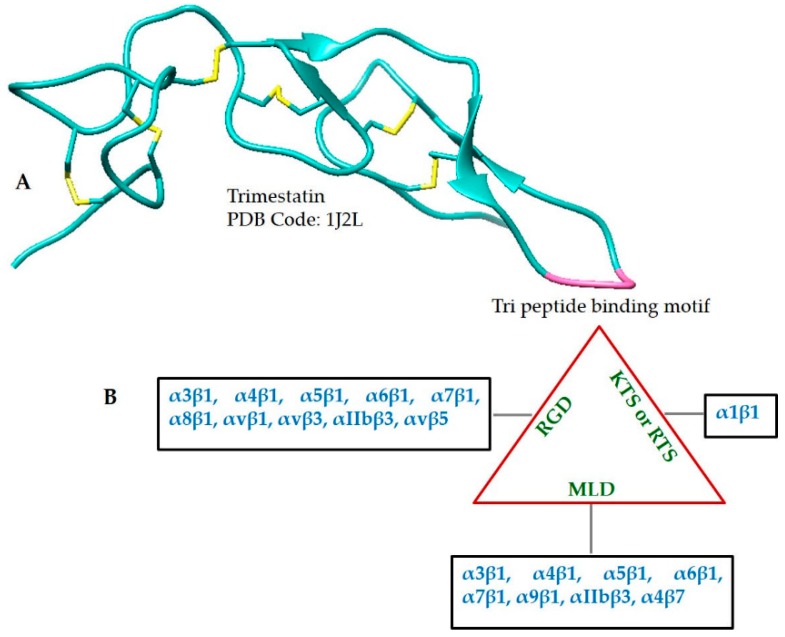
(**A**) Ribbon diagram of the crystal structure of Trimestatin (a snake venom disintegrin). The figure illustrates cysteine residues forming disulfide bonds in yellow, while the tripeptide sequence (RGD) binding motif is shown in pink. (**B**) The tripeptide sequence motifs of various snake venom disintegrins having different intermolecular interactions are shown with reference to their specificity.

Studies have shown that disintegrins are elongated peptides, consisting of several loops and turns, stabilized by disulfide bonds, as exemplified by Trimestatin shown in Figure 3A and Table 2. Disintegrins can exist as monomers, homodimers and/or heterodimers [129]. The monomers include short (49–51 amino acid residues; four disulfide bonds), medium (70 amino acids; six disulfide bonds) and long disintegrins (84 amino acids; seven disulfide bonds). Most of the disintegrins belong to the monomeric type.

Disintegrins bind to integrins, which play a fundamental role in many pathological and physiological processes. Disintegrin binding specificity with integrin is determined by the presence of a tri-peptide motif located in the hairpin loop. Therefore, three functional classes containing RGD, MLD or R/KTS motifs have been recognized. The interaction of disintegrins containing either of these motifs, with specific types of integrins is mapped in Figure 2B. Most disintegrins studied to date belong to the RGD-containing class of motifs and are monomers. Some dimeric disintegrins having a RGD subunit are also known. The MLD motif is present only in heterodimeric disintegrins. KTS/RTS-containing disintegrins selectively bind to the collagen receptor α1β1 integrin [23]. It has been suggested by different studies that the conserved aspartate residue in the tripeptide sequence of many disintegrins binds to a specific β subunit, whereas the other two amino acids determine binding affinity to the specific α integrin subunit [130].

In vivo studies showed that disintegrins containing MLD and KTF motifs are not toxic in avian and rodent models at various therapeutic doses. Further studies have shown that amino acid residues other than the tripeptide domain interact with integrin receptors. For example, any change at the C-terminus region of echistatin and eristostatin can modify their receptor binding ability. Replacement of Met^28^ with Leu selectively reduced echistatin’s ability to recognize α_5_β_1_ only. While replacement of Met^28^ with Asn completely abolished its ability to recognize integrin [131]. Recently, Urra and Araya-Maturana reviewed the anti-metastatic mechanism of snake venom toxins in detail and summarized that snake venom toxins (particularly snake venom cystatins, disintegrins, lectins, and cardiotoxins) inhibit pro-migratory and pro-invasive signals stimulated by extracellular matrix proteins and growth factors [29].

The modulatory properties of disintegrins were observed in investigations targeting various disorders, such as asthma, insulin-dependent diabetes mellitus, neurodegenerative illness, cell apoptosis, as well as, in this context, cancer angiogenesis and metastasis [132]. Saxatilin isolated and cloned from the venom of *Gloydius saxatilis*, showed thrombolytic effects in mice model. It was shown that saxatilin has no fibrinolytic activity; however, it can inhibit multiple integrins by acting on platelets [133]. A growing interest in the development of recombinant and/or chimeric disintegrins can be recognize. For example, recombinant r-mojastin and r-viridistatin (RGD-disintegrins) showed inhibition of pancreatic tumor cells. These disintegrins possess strong angiogenesis properties, and bind to ανβ_3_ and ανβ_5_ receptors. Potential applications of DisBa01 (RGD-disintegrin) have been identified, for example, also in the treatment of cancer, hernia and fibroproliferative diseases. Vicrostatin is a chimeric disintegrin which can significantly inhibit breast cancer cells and tubulogenesis [134]. Finally, disintegrins have already served as novel lead compounds supporting the design of various approved drugs, for example Aggarast and Integrilin [7,135,136].

### 2.4. Natriuretic Peptides (NPs)

Natriuretic peptides found in vertebrates play a vital role in natriuresis. Homologous peptides have also been reported in plants and bacteria. Three mammalian NPs are known: atrial natriuretic peptide (ANP), B-type natriuretic peptide (BNP), and C-type natriuretic peptide (CNP). These NPs regulate the functions of cardiovascular and renal systems, and render their function by forming a complex and binding to the natriuretic peptide receptors (NPR-A, NPR-B) [137]. For example, ANP and BNP act in an endocrine manner to maintain blood pressure and volume. Both ANP and BNP are released by cardiomyocytes in response to elevated blood pressure and hypervolemia [138,139], and CNP is produced by endothelial cells [140]. NPs are of great interest today, as they support the development of therapeutics and medical procedures for the treatment and diagnosis of unfortunate physiological conditions, such as heart failure and hypertension [7]. Further details about the structures, receptors and physiological functions can be found in recent reviews published by Volpe M and Potter L. et al. [141,142].

**Figure 4:**
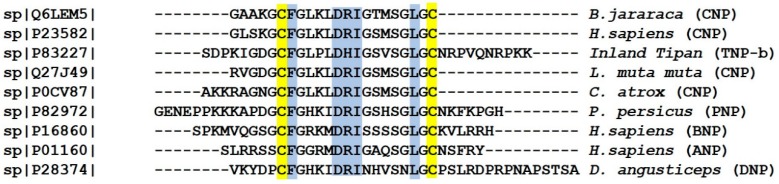
Sequence comparison of human natriuretic peptides with those of different snake venoms, aligned by Clustal W. Conserved cysteine residues are highlighted in yellow, while relatively conserved amino acid residues known for NPR binding [143] are shaded blue.

Snake venom NPs are similar to mammalian natriuretic peptides in terms of structure and function, but also possess certain distinct characteristics. All NPs have a conserved 17-residue disulfide ring and variable N- and C-termini. Five amino acid residues (F, D, R, I and L/I) within the ring structure of the NPs are crucial for receptor binding. Different NPs recognize different specific receptors by a slight sequence variation in the N- and C-terminal region [143]. Sequence alignment of different snake venom and human natriuretic peptides is shown in Figure 4. The structure of NPs is rather flexible. However, a natriuretic peptide isolated from Krait venom (known as KNP), was reported to have a putative α-helix, formed by its extended C-terminus [144]. Another study reported an *O*-glycosylated natriuretic peptide (TcNPa), from the venom of *Tropichedis carinatus*, and NMR analysis of TcNPa confirmed the presence of an α-helix as well [145].

Snake venom NPs have been reported to produce a strong hypotensive effect upon envenomation, and the prey rapidly loses consciousness [146,147]. Proteomics analysis has shown that snake venom NPs are more extensively found in Viperidae than the Elapidae. NPs constitute only 3% of the venom proteome of *Dendroaspis polyepis* and 37% of that of *Bothriechis nigroviridis* [62].

The Elapidae snake venom contains ANP/BNP natriuretic peptides and CNP natriuretic peptides are found in Viperidae. Evolutionary studies suggested that CNP natriuretic peptides were recruited independently after the divergence of vipers from more advanced snakes [148]. However, later studies reported the presence of ANP-like NPs in the venom of *C. durissus casacavella* and *C. oreganus abyssus*.

Dendroaspis natriuretic peptide (DNP) was the first natriuretic peptide isolated from the venom of *Dendroaspis angusticeps*. DNP was found to have greater stability to neutral endopeptidase than three mammalian NPs. The relatively high stability of DNP was attributed to the presence of elongated N- and C-termini [149]. The discovery of DNP led to the isolation and characterization of many novel snake venom NPs, which possess greater stability and novel mechanism of actions, as compared to their mammalian counterparts. A novel 45 amino acid mature natriuretic peptide (Na-Np) was isolated from Chinese cobra venom. This NP showed similar but relatively weak activity compared to human ANP. Also, Na-Np did not inhibit human platelet aggregation stimulated by thrombin or stejnulxin [150]. Bochra Tourki et al. studied the cardioprotective effect of Lebetin 2 (L2, isolated from the venom of *Macrovipera lebetina*) against regional and/or global ischemia-reperfusion (IR) in isolated rat hearts. Lebetin 2 is a structural homologue of BNP. In this study, it was also established that L2 utilizes identical mechanism for cardioprotection to that of BNP. The mechanism involves the activation of NP receptors and mitoK_ATP_ channels. Subsequently, mPTP (mitochondrial permeability transition pore) opening at the time of reperfusion is delayed. Consequently, IR-induced damage is attenuated. However, lebetin 2 was found to be more potent than BNP in enhancing the coronary flow, improving contractile dysfunction of myocardium and surviving global IR. It was also suggested that L2 could be a strong drug candidate for acute myocardial ischemia [151].

Recently, the development of chimeric natriuretic peptides has been the subject of interest, to combine the benefits of several peptides and avoid unwanted side effects. An interesting example in this context is Cenderitide (CD-NP), which was designed by fusing 22 amino acids of CNP with 15 amino acid residues of the carboxyl termini of DNP. Thus, CD-NP was designed to co-activate the two particulate guanylyl cyclase (pGC) receptors namely pGC-A and pGC-B. In vivo studies revealed that CD-NP, like ANP, BNP, and DNP (but unlike CNP) possesses renal-enhancing actions through pGC-A/cGMP activation. CD-NP was under clinical trials to treat cardiorenal syndrome [41]. However, Capricor Beverly Hills, California, recently completed an additional study with patients having stable heart failure in combination with moderate renal impairment. These clinical investigations performed by Capricor were terminated only recently [6].

A structure-function analysis was carried out on a NP (namely KNP) isolated from Krait venom. It was shown that KNP has two pharmacophores, a K-ring and the helix extension at the C-terminus. Both segments induce vasodilation through orthogonal pathways. Furthermore, this study also indicated the amino acids that act as molecular switches, controlling the activity of NP on smooth muscle and kidney. Thus, it can be concluded that engineered NP analogues may serve as key therapeutic leads that could help to regulate either blood pressure or volume in distinct cohorts of heart failure (HF) patients [143].

### 2.5. Bradykinin Potentiating Peptides (BPPs)

BPPs are small proline-rich hypotensive peptides consisting of 5–14 amino acid residues found in snake venom glands. Studies have also reported their presence in the brain, as part of C-type natriuretic peptide. These small peptides are well-known natural inhibitors of the angiotensin-converting enzyme (ACE). ACE is a zinc-containing metalloenzyme and an important component of the renin angiotensin system (RAS), a key regulator of blood pressure. ACE raises the blood pressure by converting angiotensin I to II (a potent vasoconstrictor) and by catalyzing the degradation of bradykinin (a natural vasodilator) [105,106].

BPPs were described first in the venom of *B. jararaca* as inhibitors of ACE [152]. Further investigations resulted in the approval and marketing of Captopril, which is an orally active peptidomimetic of the BPP [153]. This is the best and most well-known example of snake venom-based drug discovery investigation. Later similar compounds have been developed with fewer side effects, like enalapril, lisinopril, perindopril, etc., popularly known as ACE inhibitors [154]. Recently, we conducted a study on synthetic analogues of four BPPs isolated from *Agkistrodon bilineatus* and suggested that a net negative charge on the peptide is an important aspect to be considered for drug design towards ACE inhibition [155]. Nonetheless, further studies are required in this context.

**Figure 5:**
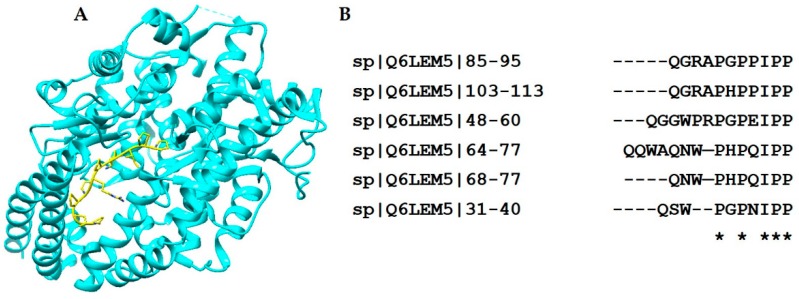
(**A**) Crystal structure of the angiotensin-converting enzyme complexed with BPP (PDB Code: 4APJ). The figure shows BPP in the channel-like cavity (i.e., active site) of the enzyme. (**B**) Sequence alignment showing the comparison of various snake venom BPPs. All peptides have a common IPP motif at the C-terminus (marked by asterisk). Left, uniprot IDs of the peptides are also provided.

Snake venom BPPs and orally active ACE inhibitors lower the blood pressure by blocking the ACE activity; as a result, the level of bradykinin in the blood increases. A large number of BPPs have been isolated from various snake venoms to date, all resembling one another in terms of structural motif, and having a proline-rich C-terminus, as shown in Figure 5. In the mature BPPs, pyroglutamate modification is usually present at the amino terminus. The modification of the N-terminus and the presence of a high percentage of proline residues are important factors in providing stability to these peptides. Structural studies of ACE in complex with a BPPb revealed that the C-terminal proline binds with the primary binding pocket of the enzyme through the formation of hydrogen bonds (Figure 5). Thus, nature has endowed these peptides with a structural motif that is highly resistant to hydrolytic degradation and that is able to penetrate the narrow channel-like active site of ACE [155].

Although all BPPs revealed high sequence similarities, their mechanism of action is not restricted to ACE inhibition only. Studies have shown that one BPP (namely, *Bj*-PRO-10c) is a potent inhibitor of ACE in vitro, but evokes a strong antihypertensive effect in an ACE-independent manner. This peptide was found to activate kidney arginosuccinate synthetase (ASS). Upon activation of ASS, the level of l-arginine and nitric oxide increases, and blood pressure decreases. *Bj*-PRO-10c was also reported to induce the release of gamma-aminobutyric acid (GABA) and glutamate by mobilizing calcium fluxes in neurons, which are also involved in controlling the blood pressure. Two other BPPs (namely, *Bj*-PRO-5a and *Bj*-PRO-10c) were demonstrated to be the modulators of bradykinin-B2 and M1 muscarinic acetylcholine receptors. These peptides induced vasodilatation in vivo. Therefore, these peptides can serve as pharmacological tools to determine new pathways and to better understand the mechanisms of blood pressure regulation. They can also help to identify novel therapeutic targets for the treatment of hypertension and related diseases [156,157].

### 2.6. Sarafotoxins (SRTXs)

Snake venom SRTXs are structurally and functionally related to vertebrate endothelin (ETs). Both families of peptides are potent vasoconstrictors and interact with endothelin receptors (ET_A_ and ET_B_), which can modulate the contraction of cardiac and smooth muscles in different tissues. SRTXs are highly toxic venomous peptides, whereas ETs are hormones produced by the mammalian vascular system [46,158]. SRTXs are exclusively and highly expressed in various isoforms in the venom gland of *Atractaspis* genus [159]. These peptides range in length from 15 to 30 amino acids as established by mass spectrometric experiments [160]. The most abundant isoform has 25 amino acids, while all other isoforms of these peptides contain a common core of 21 amino acids and adopt a highly conserved three-dimensional scaffold stabilized by an α-helical structure (Table 2). The amino acid Trp^21^ is invariably present and plays a crucial role in endothelin receptor binding [161]. It was also described in a previous study that the amino acids at positions 4–7 present a variable region of these peptides [162]. On the other hand, C-terminal extension determines the affinity and selectivity towards endothelin receptors. It was demonstrated experimentally by an in vitro study applying cloned human ET receptors that the C-terminus extension reduces the binding affinity of SRTXs to both ET_A_ and ET_B_ receptors. Furthermore, it was also suggested that steric hindrance associated with this extension was responsible for a reduced interaction between SRTX and ET receptor [161]. The respiratory effects of sarafotoxin-b (short isomer) and sarafotoxin-m (long isoform) were studied in vivo, revealing that both peptides acted differently on the respiratory mechanics. This difference of action of both peptides was also attributed to a C-terminus extension for the long isoforms. C-terminal extension can result in different spatial conformation of the two peptides [163]. SRTXs can serve to better understand the endothelin system and related diseases, such as pulmonary hypertension, asthma, and/or heart failure [163,164,165]. SRTX was used to uncover the communication pathways between the peptide and ET receptor. It was suggested that phosphoinositide second messenger system and Ca^2+^ signal-transduction mechanisms are involved in this communication [46,166,167].

### 2.7. Tripeptides

These are also very small peptides in snake venoms, consisting of only three amino acids, with pyroglutamate or Glutamate at the N-terminus and tryptophan at the C-termini [105]. They are also known to be snake venom metalloprotease inhibitors, and to date have been reported only for Viperidae snake venoms. These inhibitory peptides protect the venom gland from autodigestion by its own proteinases and also prevent the hydrolysis of other venom proteins during storage in the venom gland [168,169,170,171]. A recent study reported a synthetic tripeptide (Glu-Val-Trp) as a promising lead compound for drug design investigation with respect to restoring axonal connectivity in neurodegenerative processes [172]. Two tripeptides, namely Pt-A (Glu-Asn-Trp) and Pt-B (Glu-Gln-Trp), were purified from the venom of *Deinakistrodon acutus*. These peptides showed antiplatelet aggregation and antithrombotic effects. It was further demonstrated that both peptides protected mice from ADP-induced paralysis, but only Pt-A exerted antiplatelet aggregation activity in vivo, and not Pt-B. Therefore, it was proposed that Pt-B approaches a different mechanistic pathway to carry out its antithrombosis function from Pt-A [173].

### 2.8. Crotamine

Crotamine (myoneurotoxin) is a major component of the venom of the South American snake *Crotalus durissus terrificus* [174]. This polypeptide is composed of 42 amino acids, folded into a three-dimensional structure stabilized by three disulfide bonds, having two α-helices and two β-sheets (Table 2). The crystal structure of crotamine shows that almost all hydrophobic and charged residues are exposed to the solvent, providing a unique interaction pattern. This property of crotamine, along with a very stable structural scaffold, is considered to be responsible for its complex interaction with target proteins [175]. As a result, crotamine has demonstrated very versatile pharmacological activities. It is highly positively charged, and thus has the ability to penetrate cells, bind DNA, and can even cross the blood–brain barrier (BBB). It has also shown a particular anticancer activity against melanoma cells [176]. Crotamine is strongly attracted to cancer cells, because the surfaces of cancer cells have more negatively charged molecules compared to normal cells [177]. Studies have shown that crotamine displays analgesic properties 30 times more potent than morphine [178], and additionally possesses antimicrobial activities. Further, it can interact with different molecular targets, involved in platelet aggregation and mitochondria [179]. Crotamine also has the ability to transport drugs in mammalian cells without requiring any specific receptors. A recent study has also suggested the use of a synthetic analogue of crotamine to probe tumor cells, thus acting as a selective tool for the delivery of anticancer compounds [180]. A review published by Kerkis et al. provides details of the characteristics, properties, mechanisms of cell penetration and other biotechnological applications of crotamine [174].

### 2.9. Waprin Family

**Figure 6:**
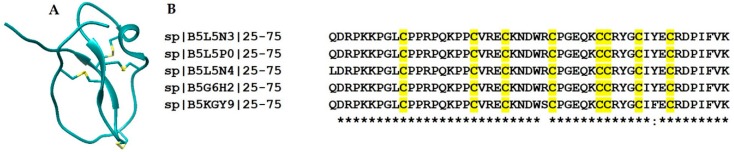
(**A**) Crystal structure of the synthesized Omwaprin peptide (PDB Code: 3NGG). (**B**) Multiple sequence alignment of the waprin-like peptides shows highly homologous primary structures. Cysteine residues are highlighted in yellow. Uniprot ID of the sequences are also provided.

Nawaprin is the first member of the ‘snake waprin family’, purified from the venom of *Naja nigricolis*. Nawaprin and omwaprin-a (from the venom of *Oxyuranus microlepidotus*) have similar functions and a distinct structural fold, resembling a flat disc-like structure with a single WAP (whey acidic protein) domain containing four disulfide bonds, short antiparallel β-sheets, a 3_10_ helix and a spiral back bone [181,182]. Figure 6A shows the structure of a synthesized omwaprin. The high sequence homology between the waprins known so far indicates that their structure is highly conserved (Figure 6B). Nawaprin bears structural similarity to elafin (a human leukocyte elastase-specific inhibitor) [182]. It has also been shown that omwaprin displays dose-dependent and strain-specific antibacterial activity and is devoid of any hemolytic activity and toxicity to mice. In the same study, the authors also concluded that the unique three-dimensional scaffold is important for the function of the peptide. Scanning electron microscopy revealed that omwaprin elicits its function through membrane disruption [183,184]. Furthermore, St. Pierre et al. proposed that Kunitz-type peptides and Waprin evolved from a common ancestral gene [185], and the transcripts of venom toxins with fused Kunitz-Wap domains were identified in the venom of *Sistrurus catenatus edwardsii* and *Suta fasciata* [186,187].

### 2.10. Waglerins

**Figure 7:**
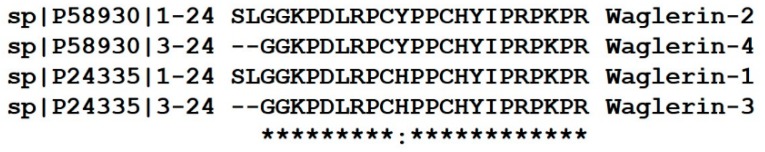
Sequence comparison of the four distinct isoforms of waglerin.

Waglerins are lethal toxins found in the venom of *Tropidolaemus wagleri*. These peptides consist of 22–24 amino acids, and four distinct isoforms have been reported to date [47,188]. These isoforms bear high sequence homology with each other and differ only by a few amino acid residues (Figure 7). A recent proteomic study revealed that Waglerin is the major toxin in the venom of *Tropidolaemus wagleri* [189], representing almost 40% of the total venom. In contrast, in a separate study, it was reported that waglerin constitutes 15% of the total composition of *Tropidolaemus wagleri* venom [190].

According to an evolutionary study conducted by Debono et al., these neurotoxic peptides are most probably the result of a de novo evolution within the pre-pro region of the C-type natriuretic peptide gene in *Tropidolaemus wagleri* venoms, a site distinct from the domain encoding for the NP [191]. These peptides mediate their neurotoxic activity by binding to the muscle nAChR. Human, mouse and rat nAChR have different specificities for Waglerin-1 (Wtx-1). It imparts the highest toxicity towards mouse and causes death by respiratory failure [192]. An anti-wrinkle cream, SYN-AKE^TM^, has been developed by Pentapharm Ltd. (Basel, Switzerland). It is a peptide mimic (H-b-Ala-Pro-DabNHB) of Wtx-1 [6]. According to Pentapharm, the Syn-Ake invokes its action by blocking the muscle nAChR in a reversible manner by blocking the ion channel. As a result, the Na^+^ uptake is substantially disturbed, and the muscles remain relaxed (http://articles.latimes.com/2009/may/03/image/ig-beauty3).

### 2.11. Antimicrobial Peptides

Cathelicidins are small cationic antimicrobial peptides (cAMP) which were reported for elapid snake venoms [193]. Some of the cathelicidins (e.g., C-BF) were shown to be able to suppress inflammation [194]. Vipercidin was reported in the venom of four South American pit vipers, and due to its excellent bactericidal profile, it could serve as promising lead molecule for the development of future peptidyl antibiotics [195].

## 3. Research Methods

Several advanced and complementary bioanalytical methods and techniques are applied to isolate, characterize and evaluate the pharmacological properties of snake venom proteins and peptides in terms of drug discovery investigations. A thorough discussion of all the methodologies is beyond the scope of this review. In brief, for investigations, snake venom is milked first, filtered through 0.45 µm filters to remove any cellular debris, lyophilized and stored at −20 °C until further use [196]. Bioassay-guided purification of the peptide is one of the most utilized methods for screening the venom in high-throughput assays against targets of therapeutic interest. This requires a combination of chromatographic methods for purifying the peptide of interest. In terms of our investigations analyzing snake venom peptides, we optimized this procedure first, as well as the further fractionation of crude venom, by applying size exclusion chromatography (SEC FPLC). We performed our purification while keeping sample solutions at pH 5.0 (0.1 M volatile buffer), which is a good choice, as most snake venoms are naturally present at pH 5.0. The bioactive fractions can then be further purified by other chromatographic modes, such as ion exchange, reversed phase and/or affinity chromatography. Recently, a new online microfluidic high-resolution screening method was employed to identify neurotoxic components from 47 snake venoms. This method provides a new analytical platform to rapidly analyze neurotoxic components of snake venom in vitro [57]. Complementary mass spectrometric methods are applied to determine molecular mass and sequence of the bioactive peptides. Since the advent of proteomics, the opportunities to discover novel bioactive peptides have accelerated substantially. During the last few years, there has been enormous development in both multidimensional chromatographic and mass spectrometric techniques. State-of-the-art mass spectrometers with high resolution and sensitivity are at the disposal of scientists, also enabling novel fragmentation techniques. Modern mass spectrometers (MS) have dual-mode ionization sources and hybrid mass analyzers to maximize performance. These days, a holistic approach, known as venomics, is employed to discover new proteins and peptides. It is an integrated approach exploiting genomics, transcriptomics and proteomics to increase the availability of information, applying only a limited amount of sample. The venom gland preparations are investigated with next-generation sequencers. These technologies have revolutionized DNA sequencing, due to the parallelization and miniaturization of the reactions. The peptides are analyzed with ESI or MALDI TOF mass spectrometers. MALDI is a very versatile source of ionization and is used in MS imaging of even the venom gland or tissue. There are two different types of MS-based sequencing approaches in use today: true de novo sequencing and data search-based sequencing. Search-based sequencing is more common, using bioinformatics tools, like Mascot (Matrix Science) or Protein Pilot (ABSciex). Different MS methods are utilized to perform MS/MS or MS^n^ or to cleave the peptide backbone, including post-source decay (PSD), collision-induced dissociation (CID) and higher collision energy dissociation (HCD). The peptides with labile posttranslational modification are analyzed with techniques based on radical fragmentation, such as electron capture dissociation (ECD) and electron transfer dissociation (ETD) [197].

Crystallographic studies of venom proteins and peptides provide insights into the three-dimensional structure and function of the individual components and its complex formation with other receptors or proteins. These studies are of utmost importance in structure-based drug discovery investigations. Many important details have already been presented to date. For example, crystallographic studies, in particular, have revealed that snake venom BPPs bind to ACE in a zinc-independent manner, while the currently available synthetic ACE inhibitor binds at the active site via a zinc metal ion [155]. Our labs at DESY, Hamburg, have the most advanced protein crystallization setups and X-ray crystallography infrastructure. We routinely perform crystallization experiments of peptides and proteins to apply crystallography in combination with highly intensive synchrotron radiation. In this context, we also developed new methods to improve protein crystallization [198,199,200]. Although realizing the highly flexible nature of some venom peptides and difficulties in crystallization, multidirectional NMR spectroscopy can be applied [201].

Apart from structural studies, different groups are working to generate large libraries of recombinant peptides by high-throughput expression of venom peptides in *E. coli* for drug discovery investigations [202,203]. Different metabolomics approaches are also used to analyze low-molecular-weight components of animal venoms [204]. In addition to these experimental approaches, bioinformatics and molecular modeling studies provide guidance for rational drug design, in understanding the complex formation of venom peptides and their receptors, and in delineating novel functions of venom peptides [205,206].

## 4. Conclusions

Snake venoms have, until now, been considered a mostly untapped resource of unique peptides and proteins. Snake venom peptides have been identified to be mostly very stable, as they have to reach the site of action before the internal mechanisms of their prey degrades, neutralizes or excretes them. Nature has met this requirement through the recruitment of highly stable molecular scaffolds, which are mostly resistant to protease degradation and can bypass other immune mechanisms of prey. Several investigations have shown that these venom peptides influence important physiological systems, such as blood pressure regulation, homeostasis, the nervous system, etc. The latest analytical technologies have made it possible to investigate several unknown snake venom peptides. These peptides can serve as molecular probes. Also, they can be used directly or as lead compounds for drug discovery and drug design investigations, because they are easy to synthesize and less prone to inducing an immune response.

## Figures and Tables

**Table 1:** Snake venom peptides with their known important biological/therapeutic applications [3,6,8,29,30].

Peptide	Mechanism of Action	Biological Significance/Therapeutics	Reference
3FTX (neurotoxin)	Selective inhibition of nAChRs at neuromuscular junction and interfere with nerve transmission.	Tool to decipher structural and functional details of nAChRs. α-cobratoxin is under clinical trial for drug-resistant HIV strains, treatment of multiple sclerosis, muscular dystrophy, myasthenia gravis and amyotrophic lateral sclerosis.	[31]
3FTX (cardiotoxin)	Membrane perturbation by electrostatic and hydrophobic interactions with the cell membranes.	Under scientific investigation for cancer inhibitory studies and potential use as anti-microbial agent.	[32,33]
Disintegrin	Selectively bind to integrin receptors present at the surface of platelet and other cells.	Tirofiban and Eptifibatide are under clinical use as antithrombotic agents. These compounds were developed from the snake venom disintegrns echistatin and barbourin. Contortrostatin is in preclinical studies for the inhibition of platelet aggregation and prostate cancer.	[34,35,36,37,38]
Kunitz-type inhibitor	Inhibition of serine proteases (e.g., plasmin, kallikrein, trypsin). Interferes with the blood coagulation cascade and fibrinolysis.	A plasmin inhibitor Textilinin-1 is in preclinical studies as antibleeding agent.	[39,40]
Natriuretic peptide	Interaction of Nps with guanylyl cyclase receptors leads to an increase of cylic guanosine monophosphate (cGMP), and affects subsequent signalling cascade. Nps can interfere the renin-angiotensin system by inhibiting the angiotensin converting enzyme.	These peptides serve as tool to understand NP biology. Cenderitide was under clinic studies for cardiovascular disease. However, its clinical development was terminated by Capiricor (US pharmaceutical company) in 2017.	[41,42,43]
BPPs	Inhibit the function of angiotensin converting enzyme, and raise the level of bradykinin.	Captopril and its analogue are under clinical use for the treatment of hypotension. These compounds were developed from the snake venom BPP.	[44]
Crotamine	Interacts electrostatically with DNA. Penetrates membranes via heparan sulphate proteoglycans binding.	Carrier for biomolecules, tool for cancer studies.	[45]
Sarafotoxin	Vasoconstriction via endothelin receptors.	Molecular probe to better understand endothelial system and related diseases.	[46]
Waglerin	nAChR antagonist.	Anti-wrinkle cosmetic cream SYN-AKE is available in the market. The active ingredient of this cream is a peptide mimic, which was designed using waglerin as a template.	[47]

**Table 2:** Snake venom peptides with well-defined three-dimensional structure.

Toxin Family	Representative Structure	Representative Sequences
**3FTX**	1UG4 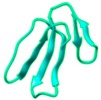	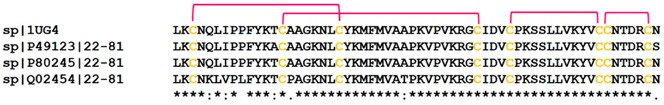
**Kunitz-type Inhibitor**	3BYB 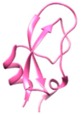	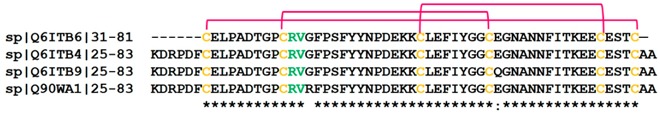
**Disintegrin**	1J2L 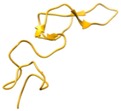	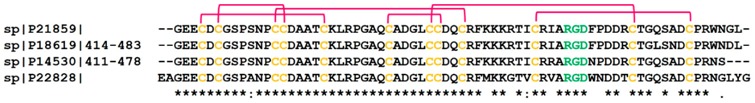
**Crotamine**	4GV5 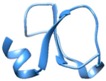	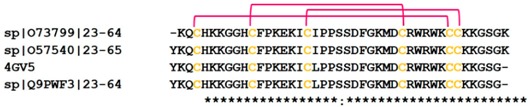
**Sarafotoxin**	2LDE 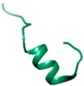	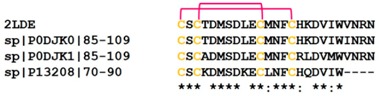

Protein Data Bank ID’s of snake venom peptide are given, along with their three-dimensional structure, in the second column. The third column presents sequence alignment prepared in Clustal W. Sequence alignment of the toxins having high homology with that of the sequence of the toxin in the second column were selected for alignment. Cysteines are colored yellow. Reactive bond residues are shown in green. The pink lines illustrate disulfide bond connectivity.
